# Electromagnetic image guidance in gynecology: prospective study of a new laparoscopic imaging and targeting technique for the treatment of symptomatic uterine fibroids

**DOI:** 10.1186/s12938-015-0086-5

**Published:** 2015-10-15

**Authors:** Donald I. Galen

**Affiliations:** Aspen Surgery Center, John Muir Memorial Hospital, Walnut Creek, CA USA; 13 Homestead Court, Danville, CA 94506 USA

**Keywords:** Fibroids, Myomas, Ablation, Electromagnetic image guidance, Acessa

## Abstract

**Background:**

Uterine fibroids occur singly or as multiple benign tumors originating in the myometrium. Because they vary in size and location, the approach and technique for their identification and surgical management vary. Reference images, such as ultrasound images, magnetic resonance images, and sonohystograms, do not provide real-time intraoperative findings.

**Methods:**

Electromagnetic image guidance, as incorporated in the Acessa Guidance System, has been cleared by the FDA to facilitate targeting and ablation of uterine fibroids during laparoscopic surgery. This is the first feasibility study to verify the features and usefulness of the guidance system in targeting symptomatic uterine fibroids—particularly hard-to-reach intramural fibroids and those abutting the endometrium. One gynecologic surgeon, who had extensive prior experience in laparoscopic ultrasound-guided identification of fibroids, treated five women with symptomatic uterine fibroids using the Acessa Guidance System. The surgeon evaluated the system and its features in terms of responses to prescribed statements; the responses were analyzed prospectively.

**Results:**

The surgeon strongly agreed (96 %) or agreed (4 %) with statements describing the helpfulness of the transducer and handpiece’s dynamic animation in targeting each fibroid, reaching the fibroid quickly, visualizing the positions of the transducer and handpiece within the pelvic cavity, and providing the surgeon with confidence when targeting the fibroid even during “out-of-plane” positioning of the handpiece.

**Conclusions:**

The surgeon’s positive user experience was evident in the guidance system’s facilitation of accurate handpiece tip placement during targeting and ablation of uterine fibroids. Continued study of electromagnetic image guidance in the laparoscopic identification and treatment of fibroids is warranted.

ClinicalTrials.gov Identifier: NCT01842789.

## Background

Uterine fibroids are the most common benign gynecologic tumors, occurring in 70–80 % of women by the time they reach 50 years of age [1]. Their negative impact on family and sexual relationships, ability to perform at work, and enjoyment of day-to-day activities has been well documented in the literature [2–4]. Because uterine fibroids can occur singly or as multiple tumors and vary in size and location, the approach and technique for their identification and surgical management also varies.

Gynecologic surgeons traditionally have identified benign fibroid tissue using reference pre-operative transvaginal ultrasound and/or sonohysterography scans, contrast-enhanced magnetic resonance images (cMRI), diagnostic hysteroscopy images, or tactile and/or visual identification at the time of surgery. Depending on the method, these approaches differ in terms of convenience, sensitivity, and range of associated direct costs from negligible (tactile identification) to costly (cMRI). In addition, reference images are static and do not provide real-time, intraoperative findings. Lack of real-time imaging is especially problematic for the surgeon if the patient has symptomatic intramural fibroids or intramural fibroids abutting—but not distorting—the endometrium (IMAEs).

In late 2012, the FDA cleared the Acessa™ System (Halt Medical, Inc., Brentwood, CA, USA) for the treatment of uterine fibroids, including hard-to-reach intramurals. The System enables the identification and targeting of uterine fibroids via laparoscopy and laparoscopic ultrasound (LUS). Under standard laparoscopy, the surgeon uses a LUS transducer to guide the radiofrequency ablation (RFA) handpiece to the edge of the target fibroid. LUS, which evidences significant sensitivity, is used to guide in two-dimensional space the insertion of the handpiece tip into the fibroid, which is subsequently ablated from the tip or from the deployed electrode array using generator settings that are based on the desired ablation zone [5–8].

In an effort to further facilitate targeting and ablation of symptomatic fibroids, particularly intramurals and IMAEs, Halt Medical recently developed (in partnership with InnerOptic Technology, Hillsborough, NC, USA) an electromagnetic image guidance system. The same electromagnetic image guidance system was previously studied and described by hepatobiliary surgeons, who attempted to target a strategically placed analog tumor in an agar block [9]. These surgeons tested their ability, time, and mental workload when targeting the analog tumor under different controlled conditions. The guidance system significantly reduced the number of required needle withdrawals and repositionings, and—once the surgeons achieved proficiency—their mental workload (as measured by the National Aeronautics and Space Administration Task Load Index) decreased significantly [9, 10]. This same system was applied to gynecologic use for targeting uterine fibroids and was cleared by the FDA to enhance the ultrasonic image of the Acessa handpiece and predict its future path on a computer monitor screen.

This feasibility study is an analysis of one gynecologic surgeon’s experience with the Acessa Guidance System when used to target, access and ablate symptomatic subserosal, intramural, IMAE, and submucosal fibroids.

## Methods

**Fig. 1:**
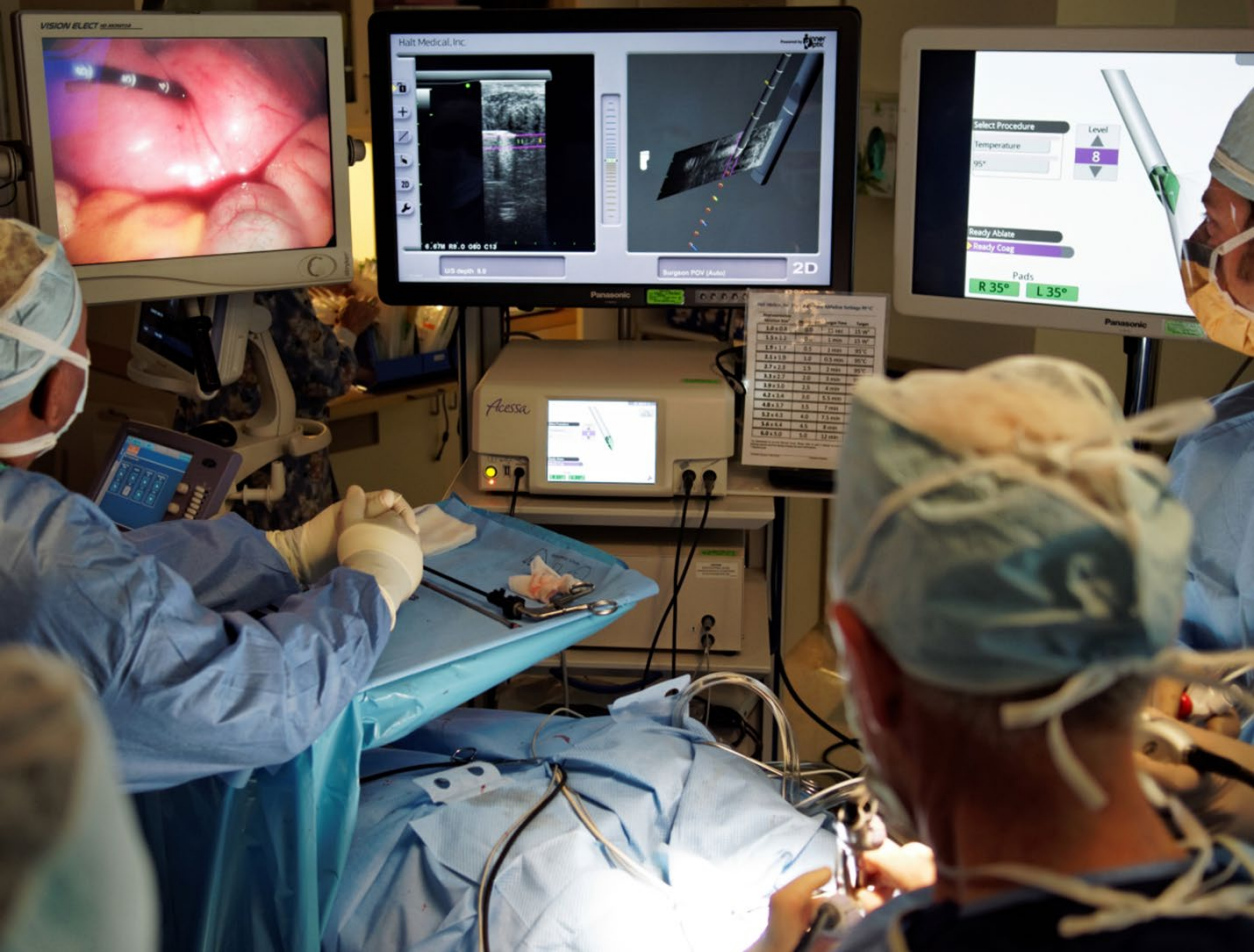
Surgeon’s view of real-time operative screens (from *left*-to-*right*): laparoscopic view of radiofrequency ablation handpiece inserted into a large intramural fibroid, Acessa Guidance screens, and Acessa System screen prior to deployment of electrode array

**Fig. 2:**
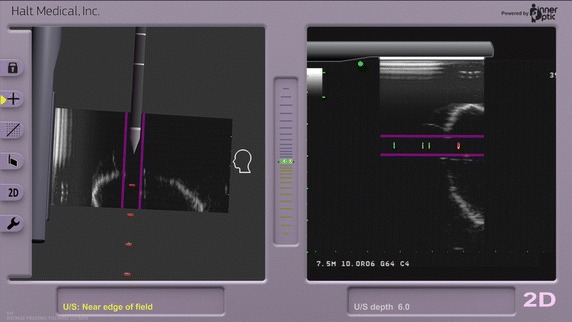
Acessa Guidance screens. (*Left*) Laparoscopic ultrasound view of Acessa handpiece tip entering fibroid from left to right within the two (*purple*) virtual tracking lines of the target zone; (*right)* computer-generated view of the relationship of the ultrasound beam and trajectory of the handpiece tip into uterus and fibroid

This ongoing study is a post-market prospective evaluation of the Acessa System with targeting animation guidance (TAG) [ClinicalTrials.gov Identifier: NCT01842789] (Figs. 1, 2). Female premenopausal subjects ≥18 years of age and with symptomatic uterine fibroids diagnosed on transvaginal ultrasound were recruited from one private gynecology office located in the San Francisco Bay Area.

The guidance system is an electromagnetic positioning system designed to guide the Acessa handpiece to the target fibroid using an overlay of the US image. A very small wire coil (sensor) is incorporated both in the tip of the Acessa handpiece and at the distal end of a sheath placed over the LUS transducer. The coil in the handpiece tip measures 0.3 mm in diameter and 11.0 mm in length; the coil at the distal end of the LUS sheath measures 1.8 × 9.0 mm. Each sensor has its own local coordinate system, which is defined by an origin and three axes and is electrically connected to the system controller. A flat field generator, placed underneath the patient’s pelvic area, generates an electromagnetic field. When the sensors are placed over the field generator, the magnetic field produced by the field generator causes the sensor coils to produce a small electrical signal. The software uses the characteristics of the induced voltage from each sensor to create virtual representations of the LUS transducer and the handpiece in relation to their spatial relationship. These avatars are displayed on an external monitor and show the physician the future path of the handpiece in relation to the displayed LUS image of the target fibroid.

After the use of the guidance system in each patient, the surgeon filled out a prescribed form to indicate his degree of agreement with statements regarding the features of the guidance system. His responses were collected prospectively and were analyzed using descriptive statistics and general trending analysis to summarize his user preference testing responses.

The local Institutional Review Board approved the study, and all participants signed informed consent.

## Results and discussion

Five female subjects (4 Caucasians and 1 African American; mean age, 42.3 ± 9.2 years of age), each of whom had 2–7 symptomatic subserosal, intramural, IMAE, and/or submucosal fibroids, were treated between July 23, 2013 and November 19, 2013 by one gynecologic surgeon. The diameters of the ablated fibroids ranged from 1.0 to 6.7 cm. The surgeon was highly experienced in LUS-guided targeting and ablation of uterine fibroids, having performed LUS-guided ablations on more than 300 fibroids prior to the study.

Despite this surgeon’s extensive experience with the standard Acessa procedure and approach (i.e., without electromagnetic image guidance), he strongly agreed (96 %) or agreed (4 %) with statements describing the helpfulness of the transducer and handpiece’s dynamic animation in targeting each fibroid, reaching the fibroid quickly, visualizing the positions of the transducer and handpiece within the pelvic cavity, and providing the surgeon with confidence when targeting the fibroid even during “out-of-plane” positioning of the handpiece (Table 1).

**Table 1:** User (physician) preference testing: summary response statistics

	Subjects, N	Mean score^a^
Targeting statements		
The transducer and handpiece dynamic animation were helpful in targeting the fibroid	5	5 ± 0
The use of TAG enabled the ability to reach the fibroid faster	5	5 ± 0
Knowing the handpiece trajectory helped to target the fibroid	5	5 ± 0
The transducer and handpiece dynamic animation helped visualize the positions of the actual transducer and handpiece in the cavity	5	5 ± 0
The use of TAG provided confidence when targeting the fibroid	5	5 ± 0
Feature statements		
The center tip-to-plane meter was useful in staying in plane	4	4.75 ± 0.5
The double green ring in the trajectory was useful in determining the location of the tip	5	3.6 ± 0.5
The purple Targeting Zone was useful in determining where the tip intersected the ultrasound plane	5	5 ± 0
While entering the fibroid and deploying the electrodes, it was easier to visualize the electrodes if the targeting zone was turned off	5	4.2 ± 0.8
The messages below the two windows provided useful information	5	3.8 ± 0.4

*TAG* targeting animation guidance

^a^Statements were evaluated according to the following scoring system: (1) strongly disagree, (2) disagree, (3) neither disagree nor agree, (4) agree, (5), strongly agree

Electromagnetic image tracking systems were initially developed in the 1990s for virtual reality and military applications [11, 12]. These systems are now widely used in the operating room in neurosurgery and otolaryngology and in vascular applications [13, 14]. The Acessa Guidance System is the first FDA-cleared electromagnetic tracking system developed for gynecologic surgery, and this is the first study to verify the system’s features and usefulness. The system does not require line of site, so the sensor coils can be imbedded near the tip of the instrument for sub-millimeter tracking accuracy. Tracking is correlated to intraoperative ultrasound and does not require registration of the patient to a previously obtained computerized tomography or magnetic resonance image.

Its significant application is in the targeting of those fibroids not readily visualized on laparoscopy (such as intramural fibroids) prior to ablation. Intramural fibroids and IMAEs have been implicated in heavy menstrual blood loss, and their ablation has been associated with reduction in bleeding over time [15]. Consequently, any technology that facilitates the targeting and treating of otherwise hard-to-reach fibroids is of benefit to the gynecologic surgeon as well as to the patient’s wellbeing.

Although this study lacks verification against a number of surgeons, it is the first and promising use in gynecology. A prospective multicenter and multi-user follow-up trial of the guidance system is planned.

## Conclusion

The Acessa Guidance System displays the real-time relationship of the movements of the handpiece and laparoscopic ultrasound transducer in the pelvic cavity. It is the first and only computer-assisted image guidance system that has been FDA-cleared for commercial use in laparoscopic surgery for uterine fibroid targeting and treatment. The positive user experience is evident in the facilitation of accurate handpiece tip placement during targeting and ablation of uterine fibroids.

## Abbreviations

cMRI: contrast-enhanced magnetic resonance imaging
IMAE: intramural fibroid abutting the endometrium
LUS: laparoscopic ultrasound
RFA: radiofrequency ablation
TAG: targeted animation guidance
US: ultrasound

## References

[1] Day Baird D, Dunson DB, Hill MC, Cousins D, Schectman JM (2003). High cumulative incidence of uterine leiomyoma in black and white women: ultrasound evidence. Am J Obstet Gynecol.

[2] Ghant MS, Sengoba KS, Recht H, Cameron KA, Lawson AK, Marsh EE (2015). Beyond the physical: a qualitative assessment of the burden of symptomatic uterine fibroids on women’s emotional and psychosocial health. J Psychosom Res.

[3] Borah BJ, Nicholson WK, Bradley L, Stewart EA (2013). The impact of uterine leiomyomas: a national survey or affected women. Am J Obstet Gynecol.

[4] Zimmermann A, Bernuit D, Gerlinger C, Schaefers M, Geppert K (2012). Prevalence, symptoms and management of uterine fibroids: an international internet-based survey of 21,746 women. BMC Women’s Health.

[5] Schirmer BD, Holzheimer RG, Mannick JA (2001). Intra-operative and laparoscopic ultrasound. Surgical treatment: evidence-based and problem-oriented.

[6] Aziz O, Ashrafian H, Jones C, Harling L, Kumar S, Garas G (2014). Laparoscopic ultrasonography versus intra-operative cholangiogram for the detection of common bile duct stones during laparoscopic cholecystectomy: a meta-analysis of diagnostic accuracy. Int J Surg.

[7] Angioli R, Battista C, Terranova C, Zullo MA, Sereni MI, Cafa EV (2010). Intraoperative contact ultrasonography during open myomectomy for uterine fibroids. Fertil Steril.

[8] Levine DJ, Berman JM, Harris M, Chudnoff SG, Whaley FS, Palmer SL (2013). Sensitivity of myoma imaging using laparoscopic ultrasound compared with magnetic resonance imaging and transvaginal ultrasound. J Minim Invasive Gynecol.

[9] Brown WL, Cassera MA, Jutric Z, Hansen PD, Hammill C. Novel device for targeting tumors in laparoscopic radiofrequency ablation: a learning curve study. in: Presented at the annual meeting of the Society of American Gastrointestinal and Endoscopic Surgeons (SAGES) Nashville, TN April 12–18, 2015.

[10] Mohamed R, Raman M, Anderson J, McLaughlin K, Rostom A, Coderre S (2014). Validation of the national aeronautics and space administration task load index as a tool to evaluate the learning curve for endoscopy training. Can J Gastroenterol Hepatol.

[11] Slater M, Usoh M, Steed A (1995). Taking steps: the influence of a walking technique on presence in virtual reality. ACM TOCHI.

[12] Nixon MA, McCallum BC, Fright WR, Price NB (1998). The effects of metals and interfering fields on electromagnetic trackers. Presence.

[13] Liu TJ, Ko AT, Tang YB, Chien HF, Hsieh TM. Clinical application of different surgical navigation systems in complex craniomaxillofacial surgery: the use of multisurface 3-dimensional images and a 2-plane reference system. Ann Plast Surg. 2015. [Epub ahead of print]. 10.1097/SAP.0000000000000429 25664409

[14] Ward TJ, Goldman RE, Weintraub JL (2013). Electromagnetic navigation with multimodal image fusion for image-guided percutaneous interventions. Tech Vasc Interv Radiol.

[15] Galen DI, Isaacson KB, Lee BB (2013). Does menstrual bleeding decrease after ablation of intramural myomas? A retrospective study. J Minim Invasive Gynecol.

